# Retinal Nerve Fiber Layer May Be Better Preserved in MOG-IgG versus AQP4-IgG Optic Neuritis: A Cohort Study

**DOI:** 10.1371/journal.pone.0170847

**Published:** 2017-01-26

**Authors:** Hadas Stiebel-Kalish, Itay Lotan, Judith Brody, Gabriel Chodick, Omer Bialer, Romain Marignier, Michael Bach, Mark Andrew Hellmann

**Affiliations:** 1 Sackler School of Medicine, Tel Aviv University, Tel Aviv, Israel; 2 Neuro-Ophthalmology Unit, Department of Ophthalmology, Rabin Medical Center, Petah Tikva, Israel; 3 Neuro-Immunology Service and Department of Neurology, Rabin Medical Center, Petah Tikva, Israel; 4 Department of Epidemiology and Preventive Medicine, Sackler School of Medicine, Tel Aviv University, Tel Aviv, Israel; 5 Service de Neurologie A, Hôpital Neurologique Pierre Wertheimer, Hospices Civils de Lyon, Lyon-Bron, France; 6 Eye Center, Medical Center, University of Freiburg, Germany, and Faculty of Medicine, University of Freiburg, Germany; Charite Universitatsmedizin Berlin, GERMANY

## Abstract

**Background:**

Optic neuritis (ON) in patients with anti-myelin oligodendrocyte glycoprotein (MOG)-IgG antibodies has been associated with a better clinical outcome than anti-aquaporin 4 (AQP4)- IgG ON. Average retinal nerve fiber layer thickness (RNFL) correlates with visual outcome after ON.

**Objectives:**

The aim of this study was to examine whether anti-MOG-IgG ON is associated with better average RNFL compared to anti-AQP4-IgG ON, and whether this corresponds with a better visual outcome.

**Methods:**

A retrospective study was done in a consecutive cohort of patients following anti-AQP4-IgG and anti-MOG-IgG ON. A generalized estimating equation (GEE) models analysis was used to compare average RNFL outcomes in ON eyes of patients with MOG-IgG to AQP4-IgG-positive patients, after adjusting for the number of ON events. The final mean visual field defect and visual acuity were compared between ON eyes of MOG-IgG and AQP4-IgG-positive patients. A correlation between average RNFL and visual function was performed in all study eyes.

**Results:**

Sixteen patients were analyzed; ten AQP4-IgG-positive and six MOG-IgG-positive. The six patients with MOG-IgG had ten ON events with disc edema, five of which were bilateral. In the AQP4-IgG-positive ON events, 1/10 patients had disc edema. Final average RNFL was significantly better in eyes following MOG-IgG-ON (75.33μm), compared to 63.63μm in AQP4-IgG-ON, after adjusting for the number of ON attacks (GEE, p = 0.023). Mean visual field defects were significantly smaller (GEE, p = 0.046) among MOG-IgG positive ON eyes compared to AQP-IgG positive ON eyes, but last visual acuity did not differ between the groups (GEE, p = 0.153). Among all eyes, average RNFL positively correlated with mean visual field defect (GEE, p = 0.00015) and negatively correlated with final visual acuity (GEE, p = 0.00005).

**Conclusions:**

Following ON, RNFL is better preserved in eyes of patients with MOG-IgG antibodies compared to those with AQP4-IgG antibodies, correlating with better visual outcomes.

## Introduction

Optic neuritis (ON) is a common inflammation of the optic nerve associated with numerous autoimmune conditions, including multiple sclerosis (MS), neuromyelitis optica spectrum disorder (NMOSD), chronic relapsing autoimmune optic neuritis (CRION) and autoimmune optic neuritis (AON)[1]. NMOSD is further subdivided into aquaporin-4 (AQP4) antibody positive disease and a seronegative form[2]. A subset of ON patients have serum IgG autoantibodies to myelin oligodendrocyte glycoprotein (MOG)[3]. Glial fibrillary acidic protein levels were elevated in the cerebrospinal fluids of patients with AQP4-IgG positive NMOSD, but absent in MOG-IgG positive cases (including those MOG-IgG positive patients who met diagnostic criteria for NMOSD), suggesting that astrocyte damage is a prominent feature of AQP4-IgG positive NMOSD[4].

Certain clinical and radiological features are suggestive of MOG-IgG positive ON, such as bilateral ON, disc edema and a predilection for the retrobulbar portion of the optic nerve[3,5]. ON in the context of MOG-IgG antibodies has been associated with a better clinical outcome than AQP4 IgG- positive ON[6,7], which commonly leaves permanent residual deficits[8].

Average retinal nerve fiber layer thickness (RNFL) correlates with visual outcome after ON[9]. The aim of this study was to examine whether MOG-IgG positive ON is associated with a better average RNFL measurement compared to AQP4-IgG positive ON, corresponding with the reported better visual outcome, after adjusting for the number of ON events.

## Patients and Methods

### Study Design

#### Standard protocol approvals, registrations, and patient consents

For this retrospective cohort study we identified patients from the database of our neurophthalmology-neuroimmunology team from 2003–2015. The study was approved by the institutional review board at the Rabin Medical Center.

We conducted the following selection process: We included all patients following AQP4-positive NMOSD-ON and MOG-positive ON seen between 2003–2015. NMOSD was defined according to the Wingerchuk et al. 2015 criteria[10]. Serum anti-MOG IgG antibodies were tested in patients with atypical ON using the live cell-based assays at the Institut d'Investigacio Biomedica August Pi I Sunyer, Barcelona, Spain. Anti-AQP-IgG antibodies were initially tested in six patients using the indirect immunofluorescence assay at the Hadassah Medical Center, Israel. However, all ten cases were later retested and confirmed using the FACS Live Cell-Binding Assay, either at the Institut d'Investigacio Biomedica August Pi I Sunyer, Barcelona, Spain, at the Neuro-immunology laboratories at Oxford or at the Mayo Clinic Medical Laboratories, USA.

Patients of both groups were admitted for acute-phase treatment during acute episodes of ON. An individualized approach was used, with high-dose intravenous methylprednisolone given to all patients during acute attacks and with the optional addition of plasmapheresis or intravenous immunoglobulins (IVIg) when needed. After the acute episode, continued care, maintenance therapy, and follow-up were provided in a step-wise escalation approach, based on individual response. Patients who elected to receive maintenance therapy were treated with rituximab, azathioprine, daily low-dose corticosteroids, IVIg, methotrexate, cyclophosphamide or mycophenolate mofetil. Maintenance treatment choice was based on a combination of patients’ preference, side effects, insurance coverage, and the perceived clinical course.

Spectral Domain (SD-HD) Cirrus OCT^®^ 4000–2713 (Cirrus HD, Carl Zeiss Meditec, Jena, Germany) was used to measure the average RNFL. Scans were obtained in adherence with the APOSTEL 9-point recommendations and the OSCAR-IB quality criteria [11,12]. The scans were done at a single site, graded by one investigator (HK), and performed in the same week as other reported measurements. Scans were obtained by two operators using a single device, in dim room light, with the patients’ pupils dilated. A volume scanning protocol was used for the optic nerve head, without eye tracking, using a 6mm cube with 200 A-scans and 200 B-scans. In addition to measuring visual acuity, a complete ophthalmological examination with funduscopy was performed within the same week. Postacquisition analysis was performed using software version 7.0.1.290 in an automated and masked fashion. Analysis was performed on eyes and the main recorded OCT outcome was the average retinal nerve fiber layer (RNFL) thickness, measured in microns (μ). Patients with insufficient documentation or without high-quality spectral-domain optical coherence data (other OCT scans; such as TD-OCT) were excluded from the analysis.

#### Outcome data collection

The following longitudinal clinical data was recorded for each patient: age, gender, inaugural event at presentation, length of follow-up (months), number of ON attacks, whether a bilateral ON attack occurred, presence of disc edema during ON, the worst visual acuity during the most severe ON attack (nadir visual acuity), final visual acuity at last follow-up, final mean visual field defect on Humphrey perimeter (in decibels (dB)), 30–2 spot size 3, (mean deviation) for each eye, the final average RNFL measurement for each eye on OCT and the ICD-10/WHO Visual Disability scale for each patient at last follow-up.

#### Statistical analysis

The generalized estimating equation (GEE) models with a Gamma distribution and log link function, using SPSS for IBM (2013) for Windows, Version 22.0. Armonk, NY: IBM Corp were used to compare the final average RNFL (as a dependent variable) between ON eyes of MOG-IgG and AQP4-IgG-positive patients, with the number of ON events as covariate. The absolute mean visual field defect (dB) and the last visual acuity (logMAR) was compared in AQP4-positive ON eyes and in MOG-positive ON eyes using GEE models. Among all study eyes, the average RNFL was correlated with mean visual field defect and with visual acuity. GEE was performed using both eyes of patients for bilateral cases controlling for within subject inter-e ye correlations.

## Results

### Demographic and Clinical Characteristics of Patients

We identified 12 patients amongst 18 with AQP4-IgG positive NMOSD who had ON, two of whom were excluded due to insufficient follow-up. We identified six patients following ON with MOG-IgG antibodies. Longitudinal data of at least 12 months following the last event was complete for all six patients with MOG-IgG ON and for ten of the 12 AQP4-IgG positive NMOSD who had ON (mean follow-up 86.5 months, minimum 15 months). Only one of the six patients with MOG-IgG positive antibodies met the criteria for NMOSD. (This patient had both ON and transverse myelitis with two extensive enhancing lesions of the cervical and thoracic spine.) All AQP4-IgG positive patients met the revised 2015 criteria for NMOSD(2). Demographic data, inaugural event and length of follow-up for each patient are detailed in Table 1. The mean age at presentation of the MOG-IgG positive patients was 41 years (range 29–52), while for AQP4-IgG positive NMOSD patients the mean age was 38 years (range 8–68). One AQP4-IgG positive NMOSD patient was eight years old at presentation, when OCT scans were not yet available. Her OCT scan was obtained between ages 15 and 22 years, four years after four optic neuritis attacks. For all other patients, the OCT was obtained at at least three months after the last ON attack resolved (both visual improvement and resolution of disc edema).

**Table 1:** Demographic data and length of follow-up for patients with optic neuritis.

Pt. No.	Gender	Antibody status	Age at presentation (years)	Initial presentation*	Follow-up (months)	Age at last OCT (years)
1	Male	MOG-IgG +	43	ON	29	45
2	Male	MOG-IgG +	39	ON	16	40
3	Female	MOG-IgG +	51	ON	15	51
4	Female	MOG-IgG +	29	TM + ON	19	29.5
5	Female	MOG-IgG +	31	ON	25	32
6	Female	MOG-IgG +	52	ON	16	52
1	Male	AQP4-IgG +	68	TM	65	73
2	Female	AQP4-IgG +	36	ON	178	51
3	Female	AQP4-IgG +	39	ON	131	50
4	Female	AQP4-IgG +	8	ON & ADEM	170	15
5	Female	AQP4-IgG +	36	TM	202	52
6	Female	AQP4-IgG +	44	ON	142	55
7	Female	AQP4-IgG +	32	ON	48	36
8	Male	AQP4-IgG +	56	ON	134	66
9	Female	AQP4-IgG + & dsDNA +	19	ON	84	26
10	Male	AQP4-IgG +	41	TM	110	39

* Inaugural event.

TM = transverse myelitis, ON = optic neuritis, ADEM = acute disseminated encephalomyelitis, dsDNA = double stranded DNA

Age at last OCT—OCT performed within a mean 3 months of the last ON event.

### Characteristics of Optic Neuritis Events and Visual Outcome

Sixteen eyes of ten AQP4-IgG positive NMOSD patients experienced 35 ON episodes (of which one was a bilateral ON, mean 3.5 events per patient), and nine eyes of six MOG-IgG positive patients had 10 ON events (of which five were bilateral, mean 1.67 events per patient). Disc edema was observed in all MOG-IgG positive ON attacks and in only one patient with AQP4-IgG antibodies. The final WHO visual disability score was normal in 4/6 MOG-IgG positive patients and mildly impaired in another 2/6. Amongst the AQP4-IgG positive patients, the final WHO visual disability score was normal in only one case. Table 2 details the number of ON attacks, the presence of disc edema during ON, the worst visual acuity (nadir) during the most severe ON event (logMAR), the final visual acuity at last follow-up, the final mean visual field defect (mean deviation) for each eye, the final average RNFL measurement for each eye and the ICD-10/WHO Visual Disability scale. Low visual acuities of counting fingers, hand motion, light perception and no light perception were recorded in accordance with the Freiburg visual acuity scale (FrACT) [13,14].

**Table 2:** Characteristics of optic neuritis attacks and outcome—functional and anatomic.

Pt.No.	No. of ON attacks	Disc edema during ON	Visual acuity at worst ON episode		Final visual acuity		Final visual field mean deviation (dB)		Final average RNFL (μm)		ICD-10/WHO Visual Disability scale
			RE	LE	RE	LE	RE	LE	RE	LE	
Patients with MOG-IgG positive optic neuritis											
1	Total—3 Bilateral—2 Left—1	+	3.00	2.00	0.0	0.0	-1.15	-0.84	60	59	Normal
2	Total—1 Bilateral—1	+	2.00	0.70	0.0	0.16	-2.99	-0.94	96	102	Mild
3	Total—2 Bilateral—2	+	2.00	2.00	0.08	0.1	-2.58	-2.11	78	64	Normal
4	Total—1 Right—1	+	0.04	0.04	0.0	0.0	-2.64	-3.62	70	82	Mild
5	Total—2 Left—2	+	0.16	0.24	0.0	0.0	-0.77	-3.1	91	60	Normal
6	Total—1 Right—1	+	0.86	0.16	0.0	0.0	-1.44	-0.32	70	72	Normal
Patients with AQP4-IgG positive optic neuritis											
1	Total—2 Left—2	-	0.0	2.00	0.06	0.16	-0.38	-11.08	79	49	Mild
2	Total—3 Right—3	-	3.00	0.7 (amblyopia)	0.06	0.54	-9.36	-3.52	54	86	Moderate
3	Total—5 Right—2 Left—3	-	0.04	0.03	0.0	0.0	-3.31	-1.42	50	52	Mild
4	Total—4 Right—1 Left—3	-	0.16	3.00	0.0	3.00	+1.11	UA	91	32	Mild
5	Total—4 Right—4	-	UA	UA	0.0	0.0	-2.80	-1.91	56	77	Normal
6	Total—5 Right—3 Left—2	-	0.16	1.38	0.16	0.1	-17.99	-11.12	45	46	Mild
7	Total—4 Right—3 Left—1	-	2.00	1.255	0.0	0.0	-1.26	-2.02	72	66	Mild
8	Total—1 Right—1	-	UA	UA	0.06	0.1	-3.90	-7.30	54	78	Mild
9	Total—4 Bilateral—1 Right—1 Left—2	+	0.04	3.00	0.0	0.06	-9.62	-7.56	60	64	Mild
10	Total—3 Right-1 Left—2	-	0.0	1.86	0.0	1.2	-0.02	Spot size V, average sensit. 300 DB	77	53	Mild

UA = unavailable, ON = optic neuritis

Disc edema occurred in all MOG-IgG associated ON events and in all ON events of patient no. 9 with AQP-IgG associated NMOSD.

### Analysis of MOG-IgG versus AQP4-IgG Antibody Status with RNFL and Visual Outcome

**Fig 1:**
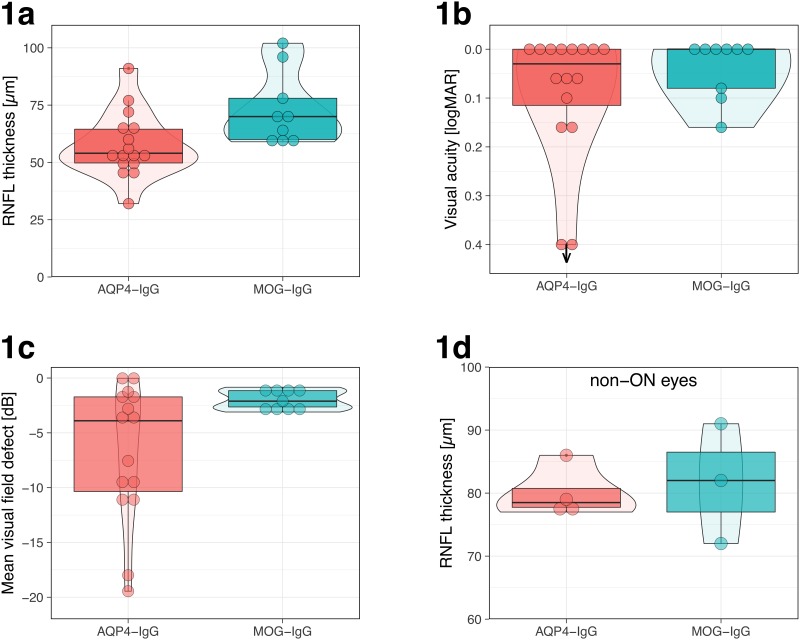
The distribution of average retinal nerve fiber layer (RNFL) thickness (1a), final visual acuity (1b) and final mean visual field defect (1c) in ON affected eyes of 16 patients following 43 optic neuritis episodes. 1d: The distribution of average retinal nerve fiber layer (RNFL) thickness in eyes *unaffected* by ON. 1b: note inverted logMAR scale: better acuity at top. The bottom two points at 0.3 logMAR with the downward arrow represent two eyes that were effectively blind. Left boxplots: Eyes of AQP4 IgG-positive patients. Right boxplots: Eyes of MOG IgG-positive patients. VA = final visual acuity. VF = mean automated Humphrey visual field defect. Box plot details: thick horizontal bar: median. Box: interquartile range (25%–75%). Whiskers: range. Dots: outliers (data >1.5 times the interquartile range off the box). The “violin plots” in the background visualize a smoothened density estimate.

RNFL thickness was significantly lower in ON eyes of AQP4-IgG positive patients (Fig 1a). The mean average RNFL measured following ON in eyes of patients with MOG-IgG antibodies was 75.33 μm (SD± 14.67), while the mean average RNFL following AQP4-IgG positive ON was 63.63 μm (SD± 14.31). The final mean visual field defect was significantly lower in ON eyes of AQP4-IgG positive patients (mean visual field defect -6.73 dB, SD ±6.3) than in post-ON eyes of patients with MOG-IgG antibodies (mean visual field defect -1.98 dB, SD ± 0.9, Fig 1c). The mean final logMAR visual acuity in ON eyes of AQP4-IgG positive patients (Fig 1b) was 0.34 (SD ±0.84) compared to 0.038 in eyes of patients with MOG-IgG antibodies (SD ±0.06). Completere data of OCT and visual data results are openly available at https://dx.doi.org/10.6084/m9.figshare.4300178.v1.

Among ON eyes, MOG-IgG was significantly associated with higher average RNFL compared to eyes of AQP-IgG positive patients, even after adjusting for number of ON attacks (GEE, p = 0.023).

The mean visual field defects were significantly smaller among ON eyes of MOG-IgG positive patients (median -2.0 dB, indicating less visual field damage), compared to ON eyes of AQP-IgG positive patients (median -3.9 dB mean visual field defect), (GEE, p = 0.046), but last visual acuity did not differ significantly between the two groups (GEE, p = 0.153).

Among all eyes, average RNFL values positively correlated with mean visual field defect (GEE, p = 0.00015) and negatively correlated with final visual acuity (GEE, p<0.0001).

## Discussion

In this cohort study of ON outcomes; average RNFL was better preserved in eyes of MOG-IgG positive patients compared to those with AQP4-IgG positive antibodies, even after adjusting for the number of ON attacks. The improved anatomical outcome in MOG-IgG positive patients closely correlated with a better visual acuity and visual field outcome.

Favorable visual outcome [15] and relative RNFL preservation (as seen in this study) may possibly be explained by the difference in the underlying autoimmune mechanism, since MOG-IgG antibodies target the myelin sheath [4,16], while AQP4-Ig-G antibodies cause an astrocytopathy [4]. Such astrocytopathy and neuroaxonal damage could explain a worse prognosis after ON in AQP4-IgG positive patients[17].

In addition to the different antibody targets; MOG-IgG optic neuropathy may be more sensitive to treatment because it involves secondary immune processes. An animal model of AQP4-IgG infused into the CSF has been shown that AQP-IgG is directly pathogenic[18]. This model suggests that some of the injury in AQP-IgG-associated NMO is a result of direct AQP4-IgG damage to the myelin and axons, without major complement activation or immune cell infiltration. The visual loss in MOG-IgG-associated injury may be more reversible due to a greater role of a secondary immune process, making it more amenable to immunosuppression, than AQP-IgG disease in which the antibody directly targets the axons and myelin [18].

A limitation of this study is the difference in disease duration between the two groups. This difference is a direct result of the fact that the first reports of MOG-associated disease [7] began almost a decade after AQP-4 antibodies were related to NMOSD [19]. Thus, the length of follow-up available for MOG-positive disease is shorter (20 mo) than that available for patients with AQP-positive NMOSD.

The results of the largest study on MOG-IgG ON to date[20,21], published in 2016, includes 50 cases of MOG-IgG positive disease and may partially contradict with ours. The authors concurr that while each episode of ON in MOG-IgG results in less optic nerve damage than single episodes of AQP4-IgG ON, the final deficits over time are comparable to those in AQP4-IgG ON, due to damage accrual driven by a higher relapse rate in MOG-IgG disease [21]. However, this study included 14 untreated attacks in 11 patients [20], which may have affected results of long-term visual outcome. Additionally, this study does not report how soon after ON symptoms did patients receive treatment, nor how closely these patients were monitored after steroid cessation for signs of relapse. We observed that MOG-IgG related ON is exquisitely steroid-sensitive and believe that time to treatment and close monitoring are important factors in visual preservation. Kleiter et al. [8], speculated that poorer visual outcome may be associated with lack of frequent short-term follow-up following an ON attack, leading to delayed escalation of therapy [8]. In each of the cases described in our report, very close short-term follow-up was maintained. For each ON attack, visual functions were monitored to ensure response after 3–5 days of high dose intravenous solumedrol, then again within a week of oral steroids, to ensure that no drop in vision occurred, and periodically every 2–6 weeks thereafter. Patients were instructed to monitor their own vision and report immediately if visual deterioration occurs. Two of the patients indeed returned earlier than planned for treatment after noting that ON relapsed when steroids were tapered. Close monitoring allows rapid escalation of therapy when needed. The small distances required for travel in Israel may facilitate close monitoring and early initial treatment with IVMP

Despite the caution needed in interpreting analyses from this small cohort, our finding are consistent with prior reports [3,22]. Whether the difference in outcomes between MOG and AQP4-IgG positive disease result from an inherent difference in the pathological mechanism of injury and response to treatment, requires further studies on the long term course of MOG-IgG positive ON and NMOSD [23].

The results of this study also highlight the fact that visual acuity alone is a poor indicator of residual deficit after ON. There was an insignificant difference in visual acuity outcome between ON eyes of MOG-IgG and AQP-IgG positive patients. However, both the mean RNFL and the mean visual field defect were better indicators of residual deficit after ON (Fig 1). We did not have complete visual evoked potential study or contrast sensitivity results to analyze whether these would be significantly different between the two groups.

We recognize the inherent statistical flaws stemming from the separate-eyed nature of our analysis, leading to a possible overstatement of statistical estimates [24]. MOG-positive optic neuritis is a rare disease, making a large-scale study of OCT findings a challenge that is not easy to overcome. We have begun to address this challenge by calling for international collaboration to pool OCT data from centers worldwide. Another approach with which we plan to correct for the difficulties in interpreting data from a rare disease is to collect and analyze repeated measures from each eye [24].

In conclusion, RNFL is better preserved in eyes following ON of patients with MOG-IgG antibodies compared to those with AQP4-IgG antibodies, correlating with visual outcome. Recognizing MOG-antibody positive associated disorders is not only an issue of nomenclature, but of potential clinical importance.
